# Supplementary material for: Analysis of somatic mutations in whole blood from 200,618 individuals identifies pervasive positive selection and novel drivers of clonal hematopoiesis
Source: Nat Genet. 2024 May 14;56(6):1147–55. doi: 10.1038/s41588-024-01755-1 (PMC11176083; doi:10.1038/s41588-024-01755-1)
Supplement: Supplementary file 1 — Supplementary Notes 1 and 2 and Fig. 1 (to go with Supplementary Note 2). [file 41588_2024_1755_MOESM1_ESM.pdf]

nature genetics

Article

https://doi.org/10.1038/s41588-024-01755-1

Analysis of somatic mutations in whole blood from 200,618 individuals identifies pervasive positive selection and novel drivers of clonal hematopoiesis

In the format provided by the authors and unedited

Supplementary notes and figures for:

Analysis of somatic mutations in whole blood from 200,618 individuals identifies pervasive positive selection and novel drivers of clonal hematopoiesis

Nicholas Bernstein[1]*, Michael Spencer Chapman[2,3]*, Kudzai Nyamondo[2,3], Zhenghao Chen[1], Nicholas Williams[2], Emily Mitchell[2,3], Peter J Campbell[2], Robert L Cohen[1]^ and Jyoti Nangalia[2,3]^

Supplementary Note 1

Our estimate of the proportion of driver mutations that have been identified follows the same logic as that used in Mitchell et al[25]. First, we estimate the total number of driver mutations within the set of coding mutations fed into the *dndscv* algorithm. This is done using the 'global dN/dS' measure i.e. a measure of the total excess of non-synonymous mutations above that expected from the number of synonymous mutations. In our case the global dN/dS is 1.13 (95% CI 1.11 - 1.16). This implies that there are an additional 13 non-synonymous mutations for every one synonymous mutation beyond that expected (ie following correction for the numbers of non-synonymous and synonymous sites respectively), which are assumed to be those under selection. The absolute number of non-synonymous drivers, $N_{DRIVERS}$, can be calculated from the total number of non-synonymous mutations in the set, N_{ACTUAL}, as follows: $N_{ACTUAL}/N_{PREDICTED}$= 1.13. But N_{ACTUAL} is a combination of non-synonymous mutations occurring by chance (which will be the same as $N_{PREDICTED}$) and non-synonymous mutations present due to selection ($N_{DRIVERS}$). Therefore:

$(N_{PREDICTED}+ N_{DRIVERS})/N_{PREDICTED}= 1 +(N_{DRIVERS}/N_{PREDICTED}) =1.13$

Rearranging this gives $N_{DRIVERS}/N_{PREDICTED} = 0.13$, ie., $N_{DRIVERS} = 0.13 * N_{PREDICTED}$

But from the original equation, $N_{PREDICTED}$ is $N_{ACTUAL}/ 1.13$, therefore, $N_{DRIVERS} = (0.13 \times N_{ACTUAL})/1.13$

In our case, N_{ACTUAL} is 39,083 and therefore $N_{DRIVERS}$ is calculated as 4,496 (95% CI 3,873 - 5,333). Importantly, this global figure includes drivers that we know about i.e. those in driver genes that we have identified, and those that we have not yet identified. Therefore, we next look at the total number of mutations within our new set of CH driver genes within this set of 39,083 non-synonymous mutations. In our case this is 2,270. Finally, we calculate the proportion of the total set of driver mutations that are now identified which is 2,270/4,496= 0.5049 = 50% (95% CI 43 -59%). The 95% confidence intervals are calculated in exactly the same way as the

median values, but using the lower and upper bounds of the original dN/dS values i.e. 1.11 - 1.16.

Supplementary note 2

To explore how the discovery of additional loci under positive selection in blood may be affected by growing sample size, we down sampled the UKBB cohort to a variety of fractions of the total cohort size, and reran *dndscv* on the reduced set of driver mutations identified to be significantly under positive selection (q<0.1). We find that for the first 200k samples, the number of genes reaching significance for positive selection for a given sample size is linear (Supplementary Figure 1). Whether this linear relationship would hold for increasing sample size requires strong assumptions about the types of mutations we may identify in a larger cohort, but increasing sample size shows no signs of asymptotic returns in driver discovery thus far. This would suggest that there is a tail of infrequently mutated genes that could reach significance thresholds in larger cohorts were their mutation numbers to be larger. Therefore, we propose that there would be merit in a substantially larger future analysis, combining different population cohorts.

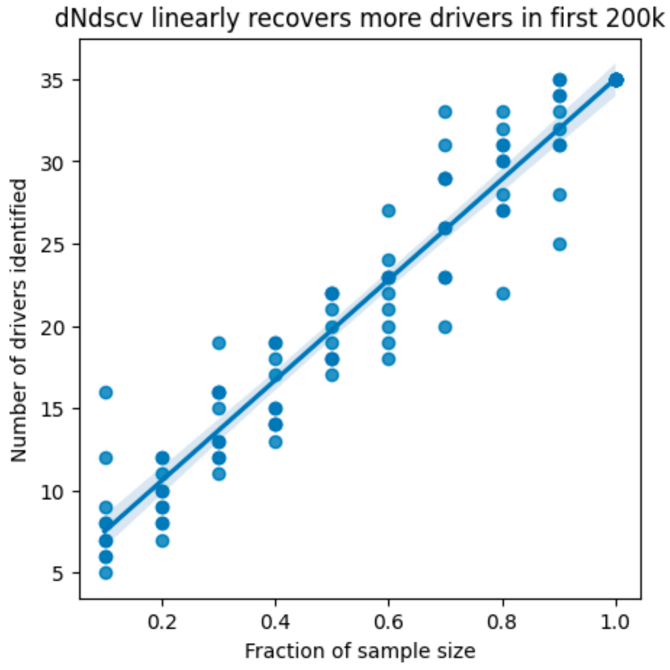

Supplementary Figure 1. *Dndscv* linearly recovers more genes under positive selection with increasing sample size. Each blue dot represents the number of genes reaching significance thresholds (q<0.1, y axis) at a particular fraction size.
