# Supplementary material for: Analysis of somatic mutations in whole blood from 200,618 individuals identifies pervasive positive selection and novel drivers of clonal hematopoiesis
Source: Nat Genet. 2024 May 14;56(6):1147–55. doi: 10.1038/s41588-024-01755-1 (PMC11176083; doi:10.1038/s41588-024-01755-1)
Supplement: Supplementary file 2 — Reporting Summary [file 41588_2024_1755_MOESM2_ESM.pdf]

nature portfolio

Corresponding author(s): Jyoti Nangalia

Last updated by author(s): 08/02/2024

Reporting Summary

Nature Portfolio wishes to improve the reproducibility of the work that we publish. This form provides structure for consistency and transparency in reporting. For further information on Nature Portfolio policies, see our Editorial Policies and the Editorial Policy Checklist.

Statistics

For all statistical analyses, confirm that the following items are present in the figure legend, table legend, main text, or Methods section.

n/a	Confirmed	
☐	☒	The exact sample size (*n*) for each experimental group/condition, given as a discrete number and unit of measurement
☐	☒	A statement on whether measurements were taken from distinct samples or whether the same sample was measured repeatedly
☐	☒	The statistical test(s) used AND whether they are one- or two-sided<br>*Only common tests should be described solely by name; describe more complex techniques in the Methods section.*
☐	☒	A description of all covariates tested
☐	☒	A description of any assumptions or corrections, such as tests of normality and adjustment for multiple comparisons
☐	☒	A full description of the statistical parameters including central tendency (e.g. means) or other basic estimates (e.g. regression coefficient) AND variation (e.g. standard deviation) or associated estimates of uncertainty (e.g. confidence intervals)
☐	☒	For null hypothesis testing, the test statistic (e.g. *F*, *t*, *r*) with confidence intervals, effect sizes, degrees of freedom and *P* value noted<br>*Give P values as exact values whenever suitable.*
☒	☐	For Bayesian analysis, information on the choice of priors and Markov chain Monte Carlo settings
☒	☐	For hierarchical and complex designs, identification of the appropriate level for tests and full reporting of outcomes
☒	☐	Estimates of effect sizes (e.g. Cohen's *d*, Pearson's *r*), indicating how they were calculated

Our web collection on statistics for biologists contains articles on many of the points above.

Software and code

Policy information about availability of computer code

Data collection	UKBiobank exome sequencing CRAM files were obtained from UKBiobank resources 23143 and 23144. Variant calling files for single cell derived haematopoietic colonies was available internally at the Sanger Institute, and for published works, also available publically as detailed in the following papers (Mitchell et al, Nature 2022, Williams et al, Nature 2022, Spencer Chapman et al, Nature 2021, Fabre et al, Nature 2022, Machado et al, Nature 2022, Spencer Chapman et al, Blood 2022. Diagnoses of individuals with haematopoietic colony sequencing were as provided by previous publications or as collected following informed consent under NHS Research Ethics Committee approval 18/EE/0199 and 07/MRE/44.
Data analysis	Mutect2 (broadinstitute/gatk:4.1.3.0) and Shearwater (v3_11, Gerstung et al, Bioinformatics 2014) were used for variant identification. 1000 genomes, ExAC and gnomAD were used to remove sequencing artefacts and common germline SNPs. Variants were called within target capture regions (UKBB resource 3801) and 100bps either side and annotated using SNPeff (v4_3) and dbSNP build GRCh38.86. Variants with features commonly associated with false positives, such as alleles only supported by the end of the read, or reads with excessive edit distance, were excluded using FINGs v1.7.1. The R package dNdScv (https://github.com/im3sanger/dndscv) was used to detect gene and global level positive selection using default settings except for the following arguments: max_muts_per_gene_per_sample = Inf, use_indel_sites=T, max_coding_muts_per_sample = Inf. COSMIC v94 (Tate et al Nucleic Acids 2019) was used to create a prior for Shearwater (v3_11). Gene set enrichment analysis and gene expression was analysed using data from Corces et al (GSE74912).

For manuscripts utilizing custom algorithms or software that are central to the research but not yet described in published literature, software must be made available to editors and reviewers. We strongly encourage code deposition in a community repository (e.g. GitHub). See the Nature Portfolio guidelines for submitting code & software for further information.

Data

Policy information about availability of data

All manuscripts must include a data availability statement. This statement should provide the following information, where applicable:

- Accession codes, unique identifiers, or web links for publicly available datasets
- A description of any restrictions on data availability
- For clinical datasets or third party data, please ensure that the statement adheres to our policy

Individual level data will be uploaded to UKBB in keeping with UKBB's data sharing agreement. Non individualised mutation data has been provided in the Supplementary Tables. Code for analyses has been made available online at https://github.com/nangalialab/UKBB_ClonalHaem_Novel_Drivers and https://github.com/mspencerchapman/Pervasive_positive_selection_in_blood.

Research involving human participants, their data, or biological material

Policy information about studies with human participants or human data. See also policy information about sex, gender (identity/presentation), and sexual orientation and race, ethnicity and racism.

Reporting on sex and gender	Sex and gender were considered in the study design - where reported gender did not match X chromosome zygosity in UKBB, samples were excluded from further analysis.
Reporting on race, ethnicity, or other socially relevant groupings	No hypotheses related to clonal haematopoiesis and race, ethnicity or socially relevant grouping were asked in this study.
Population characteristics	UKBiobank population characteristics were as previously described in Bycroft et al Nature 2018. We also analysed 10837 colonies from 50 individuals from pre-birth to >80 years of age: 10,202 colonies derived from myeloid progenitors and stem cells from healthy ageing individuals (Mitchell et al 2022, Fabre et al 2022), , cord blood (Mitchell et al Nature 2022), human foetal haematopoiesis (Spencer Chapman et al Nature 2021), individuals who have undergone allogeneic stem cell transplantation (Spencer Chapman et al, Blood 2022), individuals with myeloproliferative neoplasms (Williams et al, Nature 2022), additional datasets from additional myeloid malignancies such as therapy-related acute myeloid leukemia, chronic myeloid leukaemia and essential thrombocythaemia), and 635 B-/T-cell derived lymphoid colonies (Machado et al, Nature 2022)
Recruitment	For the UKBiobank analysis, recruitment was undertaken by UK Biobank. For the analyses of single-cell derived haematopoietic colonies from individuals with healthy haematopoiesis and haematological malignancies, we used previously published data. Individuals with sequencing data from unpublished datasets were recruited under Cambridge Blood and Stem Cell Biobank ethics, 18/EE/0199 and 07/MRE/44.
Ethics oversight	For single cell derived colonies, Ethics oversight was by the Eastern Multi-region Ethics Committee and under Cambridge Blood and Stem Cell Biobank ethics, 18/EE/0199 and 07/MRE/44.

Note that full information on the approval of the study protocol must also be provided in the manuscript.

Field-specific reporting

Please select the one below that is the best fit for your research. If you are not sure, read the appropriate sections before making your selection.

☒ Life sciences ☐ Behavioural & social sciences ☐ Ecological, evolutionary & environmental sciences

For a reference copy of the document with all sections, see nature.com/documents/nr-reporting-summary-flat.pdf

Life sciences study design

All studies must disclose on these points even when the disclosure is negative.

Sample size	The full UKBiobank dataset of 200K exomes was used for the study. For single cell derived colonies, we maximised the number of individuals and colonies based on published datasets and additional sequencing available. No sample size calculations were performed, however, we intended on using at least 10,000 single cell derived colonies (or interrogation of effectively 10,000 haematopoietic stem cells) to be sufficiently powered to detect , the dN/dS ratios observed for the additional genes in UKBB, were these mutations also to be found in the colony datasets.
Data exclusions	For UKBiobank, individuals were removed from association analyses if they had COPD, MI-Stroke, non-haematological malignancy, and haematological malignancy events prior to blood draw, and if there was a mismatch between X chromosome zygosity and reported gender. For haematopoietic colonies, all samples were considered for dn/ds analysis.
Replication	To validate mutations identified by Mutect (broadinstitute/gatk:4.1.3.0) , Shearwater (v3_11) was used for variant calling. dN/dS (dNdSc (https://github.com/im3sanger/dndscv)) was performed on both the Mutect calls, and on the intersection of Mutect and Shearwater validated calls too. Of 18 genes originally identified as under selection using Mutect for variant identification, 17 genes validated when using Shearwater. DUSP22 did not validate and was excluded from further analysis. Further validation was sought by calling identifying mutations in

	novel genes associated with clonal haematopoiesis in whole genomes of single cell derived colonies as detailed in the methods section of the manuscript.
Randomization	The analysis or study design was not randomised as no therapy was being tested as part of a clinical trial.
Blinding	Data analysis and collection was not performed blind and the investigators were not blinded to the allocation during analysis or outcome assessment.

Reporting for specific materials, systems and methods

We require information from authors about some types of materials, experimental systems and methods used in many studies. Here, indicate whether each material, system or method listed is relevant to your study. If you are not sure if a list item applies to your research, read the appropriate section before selecting a response.

Materials & experimental systems

n/a	Involved in the study
☒	☐ Antibodies
☒	☐ Eukaryotic cell lines
☒	☐ Palaeontology and archaeology
☒	☐ Animals and other organisms
☐	☒ Clinical data
☒	☐ Dual use research of concern
☒	☐ Plants

Methods

n/a	Involved in the study
☒	☐ ChIP-seq
☒	☐ Flow cytometry
☒	☐ MRI-based neuroimaging

Clinical data

Policy information about clinical studies

All manuscripts should comply with the ICMJE guidelines for publication of clinical research and a completed CONSORT checklist must be included with all submissions.

Clinical trial registration	N/A
Study protocol	The study was conducted using UK Biobank under application number 18448. Haematopoietic colony samples were collected under research ethics committee approval 18/EE/0199 and 07/MRE/44.
Data collection	Data collection was conducted by UKBiobank. For single cell derived haematopoietic colonies, no clinical data was required from participants beyond the haematological diagnosis which was gathered from the previous publications of the datasets or under Cambridge Blood and Stem Cell Biobank ethics, 18/EE/0199 nad 07/MRE/44.
Outcomes	We identified poor-health outcomes events and their corresponding dates from UKBB data as detailed in Supplementary table 10. Individuals were removed from association analyses if they had COPD, MI-Stroke, non-haematological malignancy, and haematological malignancy events prior to blood draw, and if there was a mismatch between X chromosome zygosity and reported gender. No outcomes were measured from haematopoietic colony data.
